# Cardiovascular and bone health outcomes in older people with subclinical hypothyroidism treated with levothyroxine: a systematic review and meta-analysis

**DOI:** 10.1186/s13643-024-02548-7

**Published:** 2024-05-08

**Authors:** Mia Holley, Salman Razvi, Mohammed Saif Farooq, Rosie Dew, Ian Maxwell, Scott Wilkes

**Affiliations:** 1 School of Medicine, Faculty of Health Sciences and Wellbeing, University of Sunderland, Sunderland, UK; 2 Translational and Clinical Research Institute, Newcastle University, Newcastle-Upon-Tyne, UK

**Keywords:** Subclinical hypothyroidism, Thyroid disease, Levothyroxine, Cardiovascular, Bone health

## Abstract

**Background:**

Thyroid dysfunction is common in older people, with females at higher risk. Evidence suggests that thyroid-stimulating hormone (TSH) levels naturally increase with age. However, as uniform serum TSH reference ranges are applied across the adult lifespan, subclinical hypothyroidism (SCH) diagnosis is more likely in older people, with some individuals also being commenced treatment with levothyroxine (LT4). It is unclear whether LT4 treatment in older people with SCH is associated with adverse cardiovascular or bone health outcomes.

**Methods:**

A systematic review and meta-analysis were performed to synthesise previous studies evaluating cardiovascular and bone health outcomes in older people with SCH, comparing LT4 treatment with no treatment. PubMed, Embase, Cochrane Library, MEDLINE, and Web of Science databases were searched from inception until March 13, 2023, and studies that evaluated cardiovascular and bone health events in people with SCH over 50 years old were selected.

**Results:**

Six articles that recruited 3853 participants were found, ranging from 185 to 1642 participants, with the proportion of females ranging from 45 to 80%. The paucity of data resulted in analysis for those aged over 65 years only. Additionally, a study with 12,212 participants aged 18 years and older was identified; however, only data relevant to patients aged 65 years and older were considered for inclusion in the systematic review. Of these 7 studies, 4 assessed cardiovascular outcomes, 1 assessed bone health outcomes, and 2 assessed both. A meta-analysis of cardiovascular outcomes revealed a pooled hazard ratio of 0.89 (95% CI 0.71–1.12), indicating no significant difference in cardiovascular risk between older individuals with SCH treated with LT4 compared to those without treatment. Due to overlapping sub-studies, meta-analysis for bone health outcomes was not possible.

**Conclusions:**

This systematic review and meta-analysis found no significant association between LT4 use and cardiovascular and bone health outcomes in SCH participants over 65 years.

**Systematic review registration:**

PROSPERO CRD42022308006

**Supplementary Information:**

The online version contains supplementary material available at 10.1186/s13643-024-02548-7.

## Introduction

Cardiovascular disease remains the leading cause of death worldwide, with ischaemic heart disease and stroke accounting for 8.9 million and 6.1 million deaths in 2019, respectively [1]. The United Kingdom (UK) population is increasing, and in 2018, it was predicted that by 2043, the number of people aged over 85 years will have nearly doubled to 3 million [2]. Each year, 160,000 deaths in the UK are attributed to cardiovascular events, accounting for approximately 23.9% of all deaths in the UK [3]. Several studies have found no association between subclinical hypothyroidism (SCH) and coronary heart disease, cerebrovascular, and peripheral arterial disease in the older population [4–8]. On the contrary, an association has been found between subclinical hyperthyroidism and cardiovascular risk [9, 10]. Moreover, several studies have explored the association between SCH and bone health outcomes in the ageing population, yielding inconsistent results [11–13].

Thyroid hormones are responsible for the metabolism in all tissues, including the heart, liver, brain, muscles, and bones, and thyroid hormone imbalance can lead to metabolic dysfunction [14]. The overall prevalence of hypothyroidism is approximately 5–10% in the general population in the UK, diagnosed when a person has elevated thyroid-stimulating hormone (TSH) levels [15]. SCH refers to TSH levels being higher than the accepted reference range, while free thyroxine levels remain within range [14].

Approximately 3.5% of the UK population is prescribed thyroid hormone replacement, and the number of prescriptions for levothyroxine (LT4) is increasing yearly [16, 17]. The goal of prescribing LT4 is to return the TSH level within the normal range and improve symptoms related to hypothyroidism. Since hypothyroidism is a chronic, irreversible condition, participants prescribed LT4 usually require long-term thyroid hormone treatment. The National Health and Nutrition Examination Survey (NHANES) study [18] and the Thyroid Epidemiology, Audit, and Research Study (TEARS) [19] found that serum TSH levels increase with age. The TEARS study indicated that the normal range for serum TSH could be 0.4–5.9 mU/L for participants aged 90 and over, rather than the 0.4–4.0 mU/L range currently used across all age groups in the UK [20]. The National Institute for Health and Care Excellence has recognised that TSH levels between 4.0 and 7.0 mU/L could be typical with ageing [21]. A review of cross-sectional studies estimated that nearly half the participants prescribed LT4 are either over or under-treated [22]. Both under- and over-treatment with thyroid hormones can be associated with adverse effects, particularly in older individuals, at higher risk of thyroid hormone toxicity [23].

This systematic review and meta-analysis aimed to combine the current literature on cardiovascular and bone health outcomes in SCH participants aged over 50 years to assess whether older individuals with SCH have worse cardiovascular and bone health outcomes when prescribed LT4. More specifically, this review compared the results of participants prescribed LT4 versus those untreated. This review was registered on PROSPERO, an international database of prospectively registered systematic reviews in health and social care (registration number CRD42022308006). No protocol was prepared for this review.

## Methods

### Search strategy

The Preferred Reporting Items for Systematic Review and Meta-Analysis (PRISMA) guidelines were employed in this review (Additional file 1) [24]. Two reviewers, MH and MSF, independently searched the Web of Science, Cochrane Library, MEDLINE, and EMBASE databases from inception until March 13, 2023. Any conflicts were resolved by a third reviewer, SW. Five terms were included in the search strategy based on disease, treatment, outcome event, age of participants, and study type (Additional file 2). Two reviewers, MH and MSF, removed the duplicates, and then the title and abstract of each article were screened against the inclusion criteria. The remaining articles had their full text filtered against the eligibility criteria, and their reference lists were checked for any additional qualifying studies.

### Eligibility criteria

Studies with participants aged 50 years or older diagnosed with SCH were eligible. An intervention group of participants taking LT4 and a control group of participants taking a placebo or no medication were required. Only full-text articles published in English with the study type randomised control trial (RCT), cohort study, case–control study, cross-sectional study, or longitudinal study design were considered.

Articles that included participants diagnosed with thyroid cancer, pituitary disease, secondary hypothyroidism, overt hypothyroidism, tertiary hypothyroidism, or hyperthyroidism were excluded. Also, articles including participants receiving a different form of thyroid replacement therapy to LT4 were excluded from this review. Studies examining participants exclusively with a history of cardiovascular disease or studies on pregnant females were also excluded.

### Outcome measures

Studies were required to evaluate the number of participants who experienced a cardiovascular event (ischemic heart disease, peripheral vascular disease, cerebrovascular disease, coronary angioplasty, or cardiovascular death) or experienced a bone health outcome (osteoporosis or a fragility fracture) since LT4 treatment was commenced. These two outcomes were grouped separately for synthesis. The following covariates were also considered to enable subgroup analyses: age, sex, LT4 dose, and TSH levels.

### Data extraction

Two independent reviewers, MH and MSF, screened the relevant articles against the eligibility criteria. A third reviewer (SW) resolved any conflicts. Data extraction of each study was completed, including the following details where possible:

i. Authors, title, and publication year
ii. Study period, study design, and number of participants
iii. Population demographics, e.g. age and sex
iv. LT4 dosage, frequency, and length of time prescribed
v. The number of participants experiencing a cardiovascular or bone health outcome

Any missing data were assumed to have not been collected.

The quality of studies was assessed using the Cochrane risk of bias tool for RCTs [25, 26] and the Newcastle–Ottawa scale for non-randomised trials [27]. The quality of evidence was assessed using Grading of Recommendations, Assessment, Development, and Evaluations (GRADE) guidelines [28, 29]. The assessment of both the quality of studies and the quality of evidence was independently carried out by two reviewers, MH and MSF.

### Data analysis

Statistical analysis was conducted using R, implementing the ‘metafor’ package. Heterogeneity was assessed through the *I*^2^ statistic such that if *I*^2^ = 0%, there was no heterogeneity; if *I*^2^ < 50%, there was moderate heterogeneity; if *I*^2^ > 50%, there was substantial heterogeneity [30]. Studies were pooled depending on their outcome measures—cardiovascular and bone health. The principal analysis was based on a random-effects model, pooling all suitable studies using hazard ratios (HR).

### Sensitivity analysis

A sensitivity analysis was conducted to ensure the integrity of the data. We checked for any potential duplication of participants in the studies, ensuring that each participant was counted only once.

## Results

### Search results

**Fig. 1:**
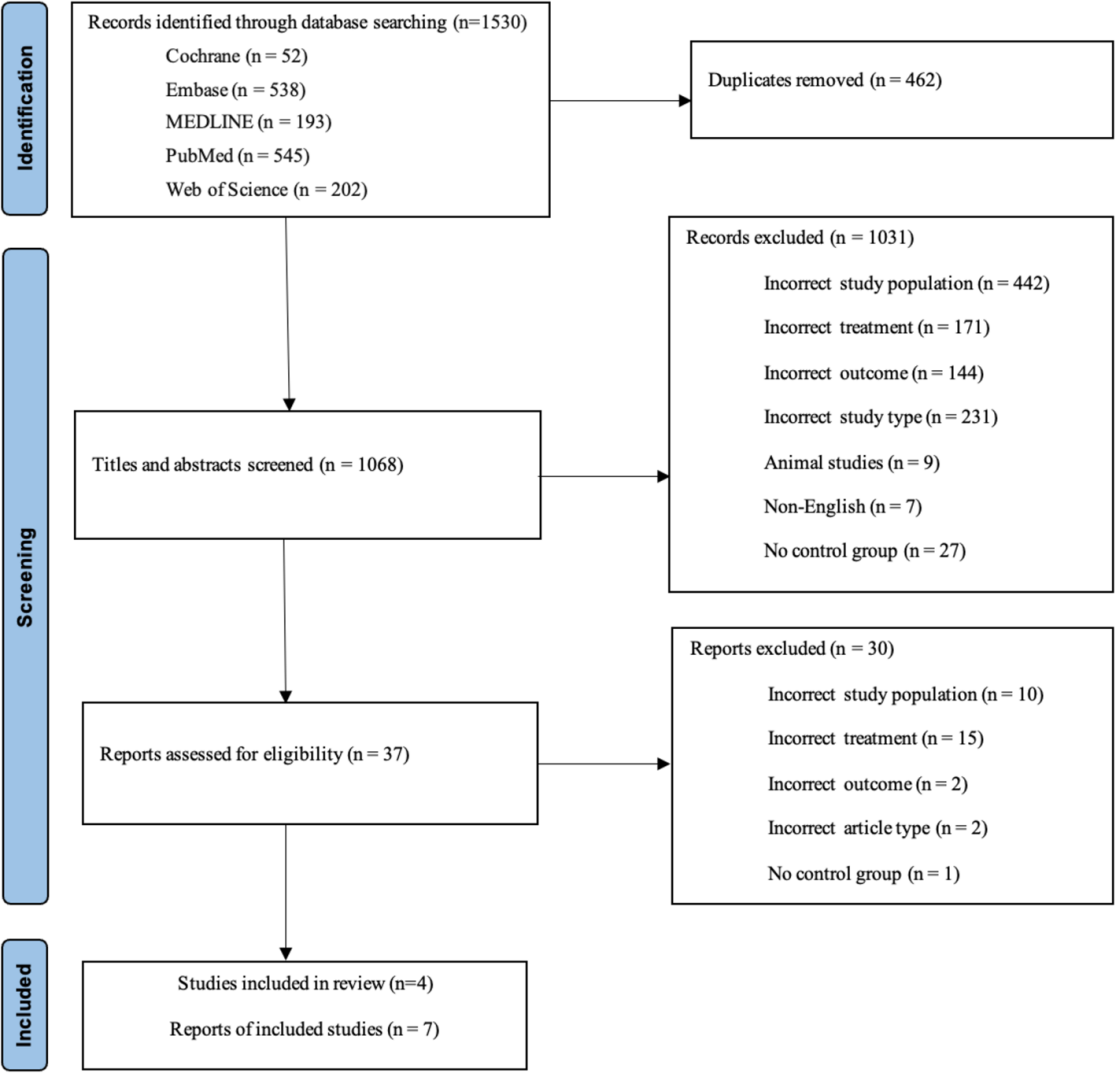
PRISMA flow diagram

There were 1530 articles identified upon the initial search (Fig. 1), with 462 duplicates. The remaining 1068 articles had their abstracts and titles screened, resulting in a further 1031 articles being excluded. The outstanding 37 articles were then fully assessed, including their reference lists [4, 7, 8, 12, 31–63]. No additional articles were found through searching reference lists. Seven articles remained in this review after reviewing the 37 articles against the specified criteria [12, 58–63]. The studies ranged from 185 to 12,212 participants. However, while one database study included 12,212 participants aged 18 years and older, it did not precisely outline the number of participants aged 65 years and over.

### Study characteristics

Two reviewers, MH and MSF, independently selected and extracted outcome data from the selected studies and their study characteristics (Table 1). Notably, one study included individuals aged 18 years and over; however, incidence risk ratios for patients aged 65 years and older were published [58]. Two studies pooled the results of the Institute for Evidence-Based Medicine in Old Age 80-plus thyroid (IEMO80 +) RCT trial (trial number NTR3851) and the Thyroid Hormone Replacement for Untreated Older Adults with Subclinical Hypothyroidism (TRUST) RCT trial (trial number NCT01660126) [59, 63]. The IEMO80 + trial included 105 participants with SCH 80 years and older; the TRUST trial included 737 participants with SCH 65 years and older. One study pooled the complete results of the two trials [59]; the other study only pooled the results of participants aged 80 years and over [63]. Two studies were nested sub-studies of the TRUST trial [12, 61]. One sub-study included 196 participants based at two of the study centres in Switzerland [12]; the other sub-study included 185 participants who underwent echocardiography [61]. Additionally, one study incorporated the results from all 737 participants in the TRUST trial [62]. No article was found publishing the data of the IEMO80 + trial individually.

**Table 1:** Study characteristics, including median levothyroxine (LT4) dose (μg/day) and mean thyroid-stimulating hormone (TSH) levels (mIU/L)

First author, year	Country	Study design	*N* (total)	*N* (treatment group)	*N* (control group)	Age of participants	Women (%)	Median LT4 dose (μg/day)	Mean TSH (mIU/L) at baseline (LT4, no LT4)
Andersen, 2015 [58]	Denmark	Retrospective database analysis	12,212^a^	2483^a^	9729^a^	≥ 65 years	79.8^a^	80	6.9^b^
Gencer, 2020 [61]	Switzerland	Randomised controlled trial	185	96	89	≥ 65years	47.0	50	6.26, 6.47
Gonzalez Rodriguez, 2020 [12]	Switzerland	Randomised controlled trial	196	100	96	≥ 65 years	45.4	50	6.3, 6.5
Mooijaart, 2019 [63]	Netherlands, Switzerland, Ireland, and the UK	Pooled results of two randomised controlled trials	251 (N1 = 146, N2 = 105)	112 (N1 = 60, N2 = 52)	139 (N1 = 86, N2 = 53)	≥ 80 years	47.0	50	6.4, 6.3
Razvi, 2012 [60]	UK	Retrospective database analysis	1642	819	823	> 70 years	80.1	75	6.77, 6.32
Stott, 2017 [62]	Netherlands, Switzerland, Ireland, and the UK	Randomised controlled trial	737	368	369	≥ 65years	53.7	50	6.41, 6.38
Zijlstra, 2021 [59]	Netherlands, Switzerland, Ireland, and the UK	Pooled results of two randomised controlled trials	842 (N1 = 737, N2 = 105)	420 (N1 = 368, N2 = 52)	422 (N1 = 369, N2 = 53)	≥ 65 years	53.2	50	6.5, 6.4

^a^Based on the total population (18 years and over)

^b^Median levels based on the total population (18 years and over)

### Study outcomes

Six studies examined cardiovascular outcomes (Table 2) [58–63]. Three studies assessed total cardiovascular events, including both fatal and non-fatal [59, 62, 63]. All three studies found a HR of less than 1, with the lowest HR found by the pooled study on participants aged over 80 years (HR 0.61; 95% confidence interval (CI) 0.24–1.50) [63]. However, all three studies had 95% CIs suggesting small sample sizes and no significant difference in cardiovascular outcomes in older SCH participants regardless of LT4 treatment. One sub-study looked at total cardiovascular events but did not calculate an adjusted HR due to the small sample size [61]. The raw outputs of the study showed no association between cardiovascular outcomes and LT4 (OR 1.09; 95% CI 0.35–3.37). The two observational database studies looked at cardiovascular mortality [58, 60]. One database study found no association between cardiovascular mortality and LT4 use (HR 1.04; 95% CI 0.56–1.93) [60]. The other database study also found no significant differences in cardiovascular outcomes regardless of LT4 use (incidence rate ratio (IRR) 1.08; 95% CI 0.88–1.34) [58]. The two observational studies had the longest follow-up time, following patients for over 3 years; the other studies had a maximum follow-up time of 3 years.

**Table 2:** Comparison of cardiovascular outcomes between patients prescribed levothyroxine (LT4) and those not prescribed LT4, including raw numbers, hazard ratios (HR), incidence rate ratios (IRR), and 95% confidence intervals (CI)

First author, year	Model adjustments	Total cardiovascular events (fatal and non-fatal)		Cardiovascular mortality events		Myocardial infarction events		Ischemic heart disease events (fatal and non-fatal)		Cerebrovascular disease events (fatal and non-fatal)	
		**LT4**	**No LT4**	**LT4**	**No LT4**	**LT4**	**No LT4**	**LT4**	**No LT4**	**LT4**	**No LT4**
Andersen, 2015 [58]	No adjustments made	–	–	100	579	41	228	–	–	–	–
		–		–		IRR (95% CI) 1.07 (0.77–1.49)		–		–	
Gencer, 2020 [61]	No adjustments made	7	6	2	0	–	–	–	–	–	–
		–		–		–		–		–	
Mooijaart, 2019 [63]	Study, country, sex, and starting dose of LT4	7	14	0	1	–	–	–	–	–	–
		HR (95% CI) 0.61 (0.24–1.50)		–		–		–		–	
Razvi, 2012 [60]	Age, sex, body mass index, total cholesterol, thyroid-stimulating hormone levels, diabetes, blood pressure, smoking status, and socioeconomic deprivation score	–	–	56	70	–	–	104	88	145	147
		–		–		–		HR (95% CI) 0.99 (0.59–1.33)		–	
Stott, 2017 [62]	Country, sex, and starting dose of LT4	18	20	2	1	–	–	–	–	–	–
		HR (95% CI) 0.89 (0.47–1.69)		–		–		–		–	
Zijlstra, 2021 [59]	Study, country, sex, and starting dose of LT4	19	25	–	–	–	–	–	–	–	–
		HR (95% CI) 0.74 (0.41, 1.35)		–		–		–		–	

Three studies examined bone health outcomes, totalling 1184 participants (Table 3) [12, 62, 63]. Among these, 2 studies looked only at fracture outcomes, and 1 looked at both fracture and osteoporosis outcomes. The study reporting on both outcomes found no association between fractures and LT4 use (HR 1.06; 95% CI 0.41–2.76) or a new diagnosis of osteoporosis (HR 0.75; 95% CI 0.17–3.37) [62]. No study found a significant difference in bone health outcomes of SCH participants aged over 65 years regardless of LT4 use.

**Table 3:** Comparison of bone health outcomes between patients prescribed levothyroxine (LT4) and those not prescribed LT4, including raw numbers, hazard ratios (HR), estimated risk difference, and 95% confidence intervals (CI)

First author, year	Model adjustments	Total bone health results		Fracture results		Osteoporosis results	
		**LT4**	**No LT4**	**LT4**	**No LT4**	**LT4**	**No LT4**
Gonzalez Rodriguez, 2020 [12]	No adjustments made	–	–	–	3	–	–
		–		–		–	
Mooijaart, 2019 [63]	No adjustments made	–	–	4	5	–	–
		–		Estimated risk difference (95% CI) 0.00 (− 0.04–0.03)		–	
Stott, 2017 [62]	Country, sex, and starting dose of LT4	12	12	9	8	3	4
		–		HR (95% CI) 1.06 (0.41–2.76)		–	

### Quality assessment

Each bias type was categorised as low, moderate, or high in line with the Cochrane Risk of Bias Tool for all five RCT studies (Additional file 3). Since the five studies incorporated two RCTs, the risk of bias was similar for all studies. Three studies were classified as low risk [12, 61, 62] and two as moderate risk [59, 63]. Non-randomised trials were given seven or eight stars on the Newcastle–Ottawa scale (Additional file 4). One study experienced quality reduction due to the non-exclusion of participants with cardiovascular problems at baseline [58]. GRADE quality of evidence assessment found a high quality of evidence for six studies (Additional file 5). The remaining study had a moderate overall quality of evidence due to a moderate risk of bias and serious imprecision [63]. Given the paucity of articles (*n* < 10), publication bias was not investigated in this systematic review [25, 64].

### Meta-analysis

**Fig. 2:**
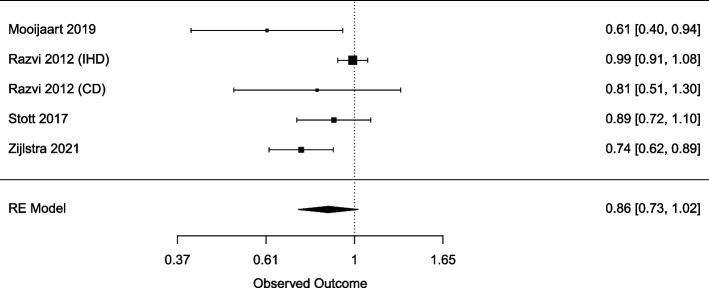
Forest plot of all studies for the association of levothyroxine (LT4) with cardiovascular effects in subclinical hypothyroid (SCH) participants, presented by hazard ratios (HR) and 95% confidence intervals (CI)

A meta-analysis was conducted for the primary outcome. The pooled HRs of the four studies are presented, of which one study calculated two HRs for two different outcome events. The pooled HR found no association between LT4 use and cardiovascular outcomes in 3668 SCH participants over 65 (pooled HR 0.86; 95% CI 0.73–1.02) (Fig. 2). No heterogeneity existed between the six articles investigating cardiovascular outcomes (*I*^2^ = 0%).

**Fig. 3:**
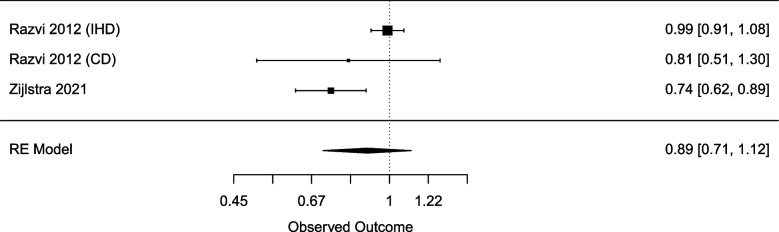
Forest plot of mutually exclusive studies for the association of levothyroxine (LT4) with cardiovascular effects in subclinical hypothyroid (SCH) participants, presented by hazard ratios (HR) and 95% confidence intervals (CI)

Due to the overlap between studies, a second analysis was carried out. The second analysis included data from one database study and one pooled study, which reported on the outcomes of all 737 participants in the TRUST trial and 105 participants in the IEMO80 + trial to exclude overlapping participants [60, 62]. This analysis of 2484 participants found no association between LT4 use and cardiovascular outcomes (pooled HR 0.89; 95% CI 0.71–1.12) (Fig. 3). No heterogeneity existed between the two articles (*I*^2^ = 0%).

A meta-analysis for the secondary outcome was not conducted as the three articles that investigated bone health outcomes all used data from the TRUST study, including the complete TRUST study and two sub-studies derived from the TRUST study.

## Discussion

### Main findings

The cardiovascular and bone health-related outcomes for participants over 65 years and prescribed LT4 remain inconclusive. There was a paucity of studies looking at the bone health and cardiovascular outcomes of LT4 in older SCH subjects. In particular, no studies were found on participants between 50 and 65 years old. The meta-analysis showed no association between adverse cardiovascular outcomes and LT4 use or not, in SCH participants over 65 years, and identified a gap in the literature for LT4 outcomes in participants with SCH between the ages of 50 and 65 years.

### Strengths and limitations

This is the largest systematic review and meta-analysis to date. The main limitation of this systematic review was the lack of suitable studies with large sample sizes and adequate power, particularly looking at bone health outcomes. This review could not find any articles about participants aged 50 to 65 years, and it identified just one article regarding osteoporosis outcomes. Only two studies had a follow-up period over 3 years, limiting the assessment of the long-term effects of LT4. Most studies included in the review had few participants or poor recruitment uptake, as demonstrated in the TRUST RCT [62]. Furthermore, this systematic review was constrained by the small number of events in all the studies considered, except Razvi et al. [60] and Andersen et al. [58] as well as insufficient raw data on the IEMO80 + trial. The results of the IEMO80 + trial were shared in a publication that included combined findings from the TRUST study, both with participants aged 65 years and over and with participants aged 80 years and over. This limited the meta-analysis as no adjusted risk estimate could be calculated individually for the IEMO80 + study. Nonetheless, the findings of all included studies are similar regardless of individual and pooled results.

### Comparison with literature

The broader literature concludes that patients prescribed LT4 who have TSH levels above 10 mIU/L and are middle-aged or young adults have better cardiovascular outcomes [65]. Clinical practice guidelines for LT4 prescribing remain unchanged, indicating LT4 for adults when they have two TSH readings above 10 mIU/L at least 3 months apart [65]. The TRUST study identified challenges in conducting an RCT to provide guidance for LT4 prescribing for this group of patients, and a large epidemiological database study may provide further evidence.

### Implications for clinical practice

This study represents the largest systematic review and meta-analysis to date and demonstrates no difference in cardiovascular health for older people with SCH whether LT4 treatment was initiated or not. The available data identified in this systematic review and meta-analysis lacks the power to give any new recommendations on prescribing LT4 for participants over 65 years with SCH. Prescribing for elderly patients with SCH will likely remain in equipoise with patient symptoms driving clinical practice.

### Supplementary Information

Supplementary Material 1: (1) PRISMA checklist. (2) Search strategy. (3) Cochrane Risk of Bias Tool for all five RCT studies. (4) Newcastle-Ottawa Scale. (5) GRADE quality of evidence assessment.

## Data Availability

This systematic review relies exclusively on the information from previously published studies, with no analysis of raw data conducted in the current study.

## Abbreviations

CI: Confidence interval
GRADE: Grading of Recommendations, Assessment, Development, and Evaluations
HR: Hazard ratio
IEMO80+: Institute for Evidence-Based Medicine in Old Age 80-plus thyroid
IRR: Incidence rate ratio
LT4: Levothyroxine
NHANES: National Health and Nutrition Examination Survey
OR: Odds ratio
PRISMA: Preferred Reporting Items for Systematic Review and Meta-Analysis
RCT: Randomised controlled trail
SCH: Subclinical hypothyroidism
TEARS: Thyroid Epidemiology, Audit, and Research Study
TRUST: Thyroid Hormone Replacement for Untreated Older Adults with Subclinical Hypothyroidism Trial
TSH: Thyroid-stimulating hormone
UK: United Kingdom
